# Ghrelin, Sleep Reduction and Evening Preference: Relationships to CLOCK 3111 T/C SNP and Weight Loss

**DOI:** 10.1371/journal.pone.0017435

**Published:** 2011-02-28

**Authors:** Marta Garaulet, Carmen Sánchez-Moreno, Caren E. Smith, Yu-Chi Lee, Francisco Nicolás, Jose M. Ordovás

**Affiliations:** 1 Department of Physiology, Faculty of Biology, University of Murcia, Murcia, Spain; 2 Jean Mayer U.S. Department of Agriculture Human Nutrition Research Center on Aging, Tufts University School of Medicine, Boston, Massachusetts, United States of America; 3 Department of Epidemiology, Population Genetics Centro Nacional de Investigaciones Cardiovasculares (CNIC), Madrid, Spain; 4 Department of Nuclear Medicine, Arrixaca Hospital, Murcia, Spain; Paris Institute of Technology for Life, Food and Environmental Sciences, France

## Abstract

**Background:**

*Circadian Locomotor Output Cycles Kaput* (*CLOCK*), an essential element of the positive regulatory arm in the human biological clock, is involved in metabolic regulation. The aim was to investigate the behavioral (sleep duration, eating patterns and chronobiological characteristics) and hormonal (plasma ghrelin and leptin concentrations) factors which could explain the previously reported association between the *CLOCK 3111T/C* SNP and weight loss.

**Methodology/Principal Findings:**

We recruited 1495 overweight/obese subjects (BMI: 25–40 kg/m^2^) of 20–65 y. who attended outpatient obesity clinics in Murcia, in southeastern Spain. We detected an association between the *CLOCK 3111T/C* SNP and weight loss, which was particularly evident after 12–14 weeks of treatment (*P* = 0.038). Specifically, carriers of the minor C allele were more resistant to weight loss than *TT* individuals (Mean±SEM) (8.71±0.59 kg *vs* 10.4±0.57 kg) C and TT respectively. In addition, our data show that minor C allele carriers had: 1. shorter sleep duration Mean ± SEM (7.0±0.05 vs 7.3±0.05) C and TT respectively (*P* = 0.039), 2. higher plasma ghrelin concentrations Mean ± SEM (pg/ml) (1108±49 *vs* 976±47)(*P* = 0.034); 3. delayed breakfast time; 4. evening preference and 5. less compliance with a Mediterranean Diet pattern, as compared with *TT* homozygotes.

**Conclusions/Significance:**

Sleep reduction, changes in ghrelin values, alterations of eating behaviors and evening preference that characterized *CLOCK 3111C* carriers could be affecting weight loss. Our results support the hypothesis that the influence of the *CLOCK* gene may extend to a broad range of variables linked with human behaviors.

## Introduction

The current prevalence of obesity has been attributed to changes in our diet and physical activity; however, recent research has raised interest in the possibility that changes in our daily behavioral patterns may be a significant contributing factor [1]. Thus, in industrial countries, the amount of daily sleep has declined by 1.5 h over the past century, concurrent with a significant increase in obesity [2]. Although these observed correlations do not support a causal relationship, additional evidence based on data from night-shift workers and sleep-restricted subjects supports the notion that [3] a sleep disruption and therefore, modification of the circadian rhythm, may play a significant role in the etiology of obesity.

*Circadian Locomotor Output Cycles Kaput (CLOCK*), an essential element of the positive regulatory arm in the human biological clock, is involved in metabolic regulation [4]–[7]. Therefore, *CLOCK* disruptions may affect the homeostasis of different metabolic pathways. Along these lines, we previously demonstrated associations between the *CLOCK 3111T/C* (rs1801260) single nucleotide polymorphism (SNP), baseline body weight and weight reduction in obese patients participating in a Mediterranean diet based weight reduction program [8]. Allele specific behavioral habits in sleep and food intake may be responsible for these effects. Indeed, previous studies have shown that this particular SNP may be associated with sleep disorders in subjects affected by major depressive and bipolar disorders [9].

The specific mechanisms driving these effects are unknown; however, these could be mediated through ghrelin and leptin; both follow circadian rhythmicity and are implicated in food intake. Ghrelin stimulates appetite and recent evidence suggests that ghrelin affects sleep [10], [11]. This is consistent with the notion that the neurophysiologic and metabolic mechanisms responsible for the control of food-seeking and sleep behaviors, are coordinated, thus pairing hunger and vigilance during the daylight and satiety and sleep during darkness [12].

The aims of the present study were to evaluate behavioral and chronobiological aspects of resistance to weight loss associated with the *CLOCK 3111T/C* SNP in an obese, mostly sedentary population. Specifically, we investigated sleep duration, eating behaviour and circadian characteristics as well as ghrelin and leptin plasma values as possible markers of sleep and intake-related processes.

## Materials and Methods

### Subjects and methods

We recruited overweight or obese subjects (BMI>25 kg/m^2^ and <40 kg/m^2^) within the age range of 20–65 years, (n = 1495). Most of the population studied were females (82.5%). Patients attended five outpatient obesity clinics from 2009–2010 in the city of Murcia, located in southeastern Spain. Patients receiving thermogenic or lipogenic drugs, or those diagnosed with diabetes mellitus, chronic renal failure, hepatic diseases or cancer were excluded from the study. 1290 individuals were genotyped for the *CLOCK 3111T/C* SNP. All procedures were in accordance with good clinical practice. Written consent was obtained from each patient before participation and the study protocol was approved by the Research Ethics Committee of the Virgen de la Arrixaca Hospital. Patient data were codified to guarantee anonymity.

### Characteristics of the treatment

The characteristics of the weight reduction program (Garaulet method©) have been described elsewhere [13], [14]. Briefly, during the initial months, subjects attended a weekly 60-min therapy session in support groups (n = 10), followed by a 5-month maintenance period. Sessions were conducted by a nutritionist. Treatment was based on the following: Dietary treatment. Dietary individual energy requirements were assessed by calculating [1] resting energy expenditure (REE) according to the Harris-Benedict formula and [2] total energy expenditure (TEE) according to the type and duration of physical activity estimated by the International Physical Activity Questionnaire (IPAQ). Next, about 600 kcal per day were subtracted from the TEE. The final dietary energy content ranged from 1200–1800 kcal per day for women and 1500–2000 kcal per day for men to induce an approximate loss of 0.5–1 kg per week. The recommendations were consistent with the Mediterranean type of diet [13], [14] and the macronutrient distribution followed the recommendations of the Spanish Society of Community Nutrition [15]. Nutritional education was given during group therapy sessions to help subjects plan their own menus and to educate subjects to adopt appropriate lifetime eating habits. Physical activity emphasized individual goals of 15–30 min or more of moderate intensity physical activity, at least 2 or 3 times a week. Patients were encouraged to use a pedometer to reach at least 10 000 steps per day. Behavioral techniques included stimulus control, self-monitoring, positive reinforcement and cognitive behavioral therapy.

### Anthropometric and biochemical measurements

Subjects were weighed barefoot wearing light clothes, with a digital scale to the nearest 0.1 kg, at the same time each day weekly to assess weight loss during treatment. Height was measured using a Harpenden digital stadiometer (rank 0.7–2.05). The subject was positioned upright, relaxed and with the head in the Frankfurt plane. BMI was calculated according to these measurements as weight (kg)/(height(m))^2^. Total body fat was measured by bioelectrical impedance using TANITA TBF-300 (TANITA Corporation of America, Arlington Heights, IL, USA) equipment. Body fat distribution was assessed by the measurement of different circumferences: waist circumference, at the level of the umbilicus, and hip circumference, as the widest circumference over the greater trochanters [16]. All measurements were made with a flexible and inextensible tape measure.

### Ghrelin and leptin

Fasting blood samples, collected at 08:00AM were centrifuged at 4°C, and the plasma was stored at −70°C for subsequent analysis. Plasma leptin and ghrelin samples were measured by radioimmunoassay (Linco Research, St. Charles, MO). All samples for leptin and ghrelin were run in duplicate.

### Sleep Hours

Habitual sleep time was estimated by a questionnaire. ‘During week days: How many hours (and minutes) do you usually sleep? and; ‘During weekend days: How many hours (and minutes) do you usually sleep? A total weekly sleep score was calculated as: ((min Weekdays x 5) + (min Weekend days x 2))/7.

### Morning-evening Questionnaire

Subjects completed the Morningness/eveningness (M/E) questionnaire (MEQ) 19 item scale of Horne and Ostberg, 1976 [17]. M/E typology is a way to characterize subjects depending on individual differences of wake/sleep patterns and the time of day people feel or perform best. Some people are night “owls” and like to stay up late at night and sleep late in the morning (Evening type), while others are early birds and prefer to go to bed at an early hour and arise with the break of dawn (Morning types). The majority of people are in between and categorized as “Neutral types” [18]. Evening types were considered as scoring under 53 and morning types above 64. All subjects within the range of 53 to 64 were classified as neutral type [18].

### Habitual dietary intake

To evaluate food habits, initial nutrient intake was determined by a 24-h dietary recall. Interviews were conducted from Monday to Friday, including 24-h recalls of food intake from weekend and weekdays. Total energy intake and macronutrient composition from the initial 24-h recalls were analyzed with the nutritional evaluation software program Grunumur [19], [20] on the basis of Spanish food composition tables [20]. The intakes of fatty acids were calculated from Spanish food-composition tables [21]. Over the course of one week, patients recorded the time of day that each meal (eg, breakfast, lunch, dinner) was eaten.

### Physical activity (PA) questionnaire

To assess PA within the last 7 days, the International Physical Activity Questionnaire (IPAQ), was administered with the help of a nutritionist. It was developed for adults between 18–65 years, assessing the different domains of PA (work, transport, house and garden and leisure time) and the volume of activity was computed by weighting each type of activity by its energy requirements defined in metabolic equivalents METs [22]. It is known to be a valid and reliable instrument to measure PA at the population level [23].

### DNA isolation and clock genotyping

DNA was isolated from blood samples using routine DNA isolation sets (Qiagen). We performed genotyping of *CLOCK* gene polymorphisms using a TaqMan assay with allele specific probes on the ABI Prism 7900HT Sequence Detection System (Applied Biosystems, Foster City, CA, USA) according to the standardized laboratory protocols [24].

### Statistical analyses

We used Pearson's chi-square (χ^2^) test and the Fisher test as statistical procedures to evaluate differences in frequencies. We applied ANOVA and the Student t-test to compare crude means across genotype groups. We tested different genetic inheritance models and a dominant model was applied in the final analyses. We performed multivariate adjustments of the associations by analysis of covariance and estimated adjusted means. We adjusted analyses for potential confounders including sex, age, and study site. We also tested the statistical homogeneity of the effects by sex in the corresponding regression model with interaction terms. Statistical analyses were performed using SPSS 15.0 software (SPSS). A two-tailed P-value of <0.05 was considered statistically significant.

## Results

### General characteristics

General characteristics of the population studied are shown in **Table 1**. By study design, subjects were overweight or obese and they had low levels of physical activity. On average they slept 7 hours/day with a predominance of morning type individuals. The dietary habits were consistent with lower carbohydrate and higher protein and fat intakes than those recommended in Spanish guidelines [25]. However, the type of dietary fat was consistent with the Mediterranean diet pattern (>50% of total fat quantity in grams as monounsaturated fat). The distribution of the *3111T/C* genotypes did not differ significantly from that expected according to Hardy Weinberg equilibrium (X^2^ = 1.167; *P* = 0.279). During the treatment period which included dietary advice, 81% of the population fulfilled the Mediterranean Diet recommendations.

**Table 1:** General characteristics of the population studied.

	Mean	SD
Age (yrs)	39.37	12.29
***Anthropometric***		
BMI (kg/m^2^)	31.12	5.38
Weight (kg)	84.10	17.34
Height (m)	1.64	0.08
Body fat (%)	37.34	6.63
Waist (cm)	102	15
Hip (cm)	114	10
***Weight loss***		
Weight loss (Kg)	9.01	5.61
Percentage of weight loss (%)	11.1	6.16
Treatment Weeks (n)	21	17
Rate of weight loss (g/week)	500	450
***Activity*** (n = 1204)		
Exercise (METs)	4474	7544
***Dietary intake***		
Total Energy (kcal/day)	2066.7	715.4
Proteins (%)	17.0	4.6
Carbohydrates (%)	41.8	10.6
Fats (%)	42.2	9.6
MUFA% (Total fat)	55.5	8.1
***Food-Intake Hormones***		
Ghrelin (pg/ml)	1057.81	995.3
Leptin (ng/ml)	19.60	14.11
***Chronobiological Characteristics***		
Sleep (hours *per* day)	7.23	1.25
Morning-evening-score	51.70	10.10
Morning evening classification	n	(%)
Neutral type	359	24
Morning type	628	42
Evening type	508	34
***CLOCK Polymorphism.*** (n = 1290)	n	(%)
*TT*	684	53.0
*TC*	500	38.8
*CC*	106	8.2

Abbreviations used: BMI; Body mass index, METs; Metabolic equivalents, MUFA; Monounsaturated Fatty Acids.

### CLOCK 3111T/C SNP and weight loss

**Figure 1:**
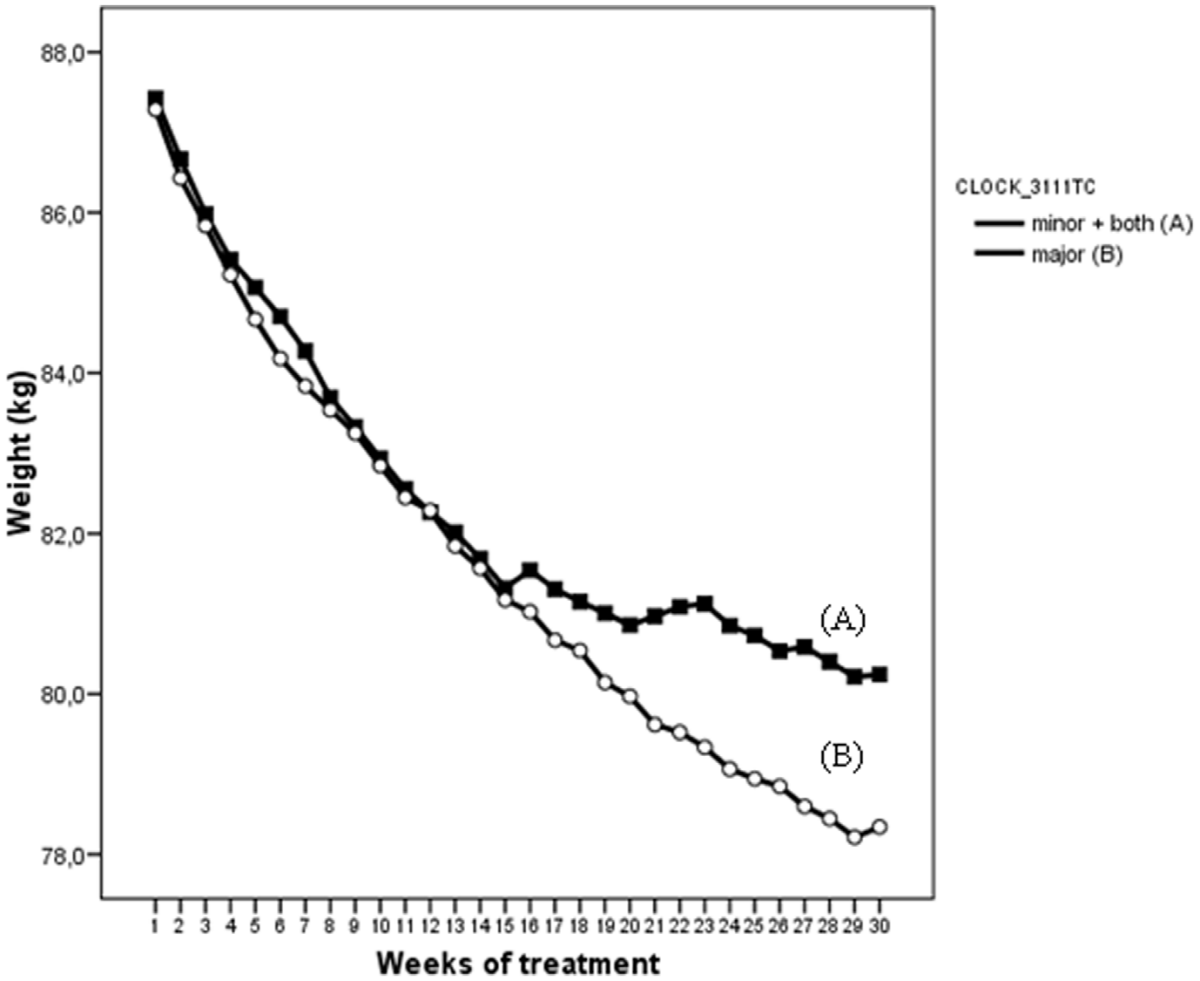
*CLOCK 3111T/C* genotype and weight loss progression during 28 weeks of treatment.

We found an association between the *CLOCK 3111T/C* SNP and weight loss. Subjects carrying the minor *C* allele lost significantly less weight (Mean±SEM) (8.71±0.59 kg) compared to *TT* subjects (10.4±0.57 kg). This effect was particularly evident after 16 weeks of treatment (*P* = 0.038) (**Figure 1**). When patients were divided into low and high groups according to median age, differences in weight loss among *C* and *TT* carriers were only significant in the older group (≥38 y.) (*P* = 0.035) while differences were not significant in the younger group (*P* = 0.297).

### CLOCK 3111T/C, ghrelin, leptin, and eating behaviors

**Figure 2:**
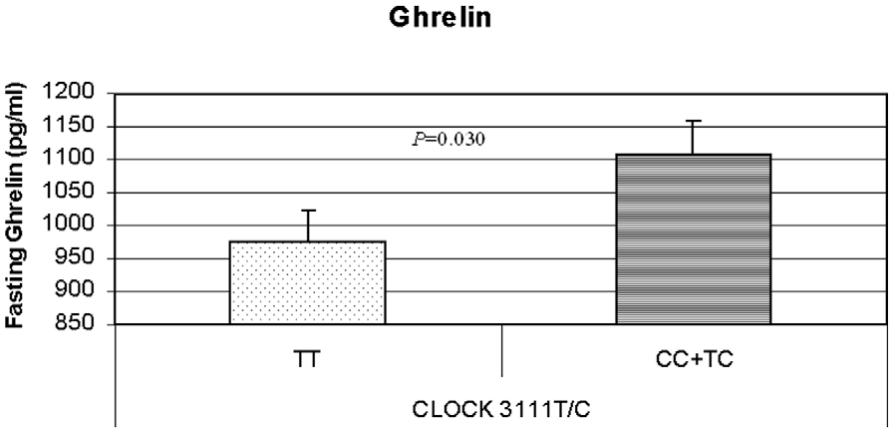
Significant associations between *CLOCK 3111T/C* and fasting ghrelin.

We obtained significant associations between the *CLOCK 3111T/C* and plasma ghrelin levels that were higher in carriers of the minor allele (*C*) than in non-carriers (*TT*) **(** **Figure 2** **)** (*P* = 0.034). The statistical association was stronger among the older patients (aged ≥38 y; *P* = 0.022). Moreover, among C carriers we also observed a negative and significant correlation between ghrelin and weight loss (r = −1.37; *P* = 0.009). However, we did not find a significant association between leptin and the *CLOCK 3111T/C* SNP.

Analysis of eating behaviors revealed a trend towards overeating at certain times of the day (*P* = 0.06) in minor allele (*C*) carriers, and these subjects also started to eat later in the morning than non-carriers, particularly during week-ends (*P* = 0.044) (10.00AM *vs.* 9:43AM, respectively).

### CLOCK 3111TC SNP and Mediterranean dietary habits

Minor *C* allele carriers showed a lower adherence to a Mediterranean Diet pattern than non-carriers (**Table 2**). Specifically they consumed a higher percent of their daily energy intake as protein (*P* = 0.032) and higher amount of trans fatty acids (*P* = 0.039). Conversely, they reported a significantly lower intake of monounsaturated fatty acids (MUFA) (*P* = 0.045). Moreover, they had a tendency to consume a higher percent as fat (*P* = 0.072) and a lower percent as carbohydrate (*P* = 0.065).

**Table 2:** Associations of *CLOCK 3111TC* SNP with dietary intake.

	*TT*		*CC+TC*		
	Mean	SEM	Mean	SEM	*P*
Total Energy (kcal/day)	2069.96	34.78	2086.91	37.63	0.741
Proteins (%)	16.58	0.23	17.29	0.24	**0.032**
g/day	84.59	1.59	87.26	1.72	0.257
Carbohydrates (%)	37.87	0.47	36.76	0.44	0.065
g/day	151.66	2.80	146.02	3.00	0.149
Fiber (g/day)	19.25	0.54	18.15	0.58	0.163
Fats (%)	45.20	0.38	46.18	0.41	0.072
g/day	86.75	1.85	96.41.	1.73	0.894
MUFA% (total fat)	56.02	0.39	54.92	0.42	**0.045**
PUFA% (total fat)	13.65	0.19	13.75	0.20	0.711
SFA% (total fat)	29.58	0.41	30.42	0.44	0.162
Trans FA (g)	0.58	0.06	0.76	0.06	**0.039**

Abbreviations used: MUFA; Monounsaturated fatty acids, PUFA; Polyunsaturated fatty acids, SFA; Saturated fatty acids.

Adjusted for sex, age and study site.

### CLOCK 3111T/C, Sleep duration, Morningness/eveningness questionnaire and physical activity

Carriers of the minor *C* allele reported shorter daily sleep duration (h) Mean ± SEM (7.0±0.051) than *TT* homozygotes (7.3±0.058) (*P* = 0.039). With respect to the Morningness/eveningness questionnaire, in response to the question “At what time of the day do you think that you reach your “feeling best” peak?” *C* carriers were more represented in the “feeling best in the evening” category than *TT* subjects who comprised a larger proportion of the ‘feeling best in the morning’ subjects (*P* = 0.007; Table 3). Specifically, 57% of the subjects feeling best in the morning were TT subjects while 43% were C carriers whereas 53% of subjects feeling best in the evening were C carriers and 47% were TT carriers. Similar findings were obtained for other morningness/eveningness related questions which supports the classification of *C* carriers as evening persons (**Table 3**). This evening preference could contribute to lower physical activity and resistance to weight loss among *C* carriers. Indeed, among the minor allele *C* carriers an inverse and significant correlation was found between evening preference (as assessed by the morning-evening-score) and physical activity (r = −0.186, *P* = 0.007).

**Table 3:** Genotype distribution of *CLOCK 3111T/C* according to Morningness/Eveningness characteristics.

	Time of the day feeling best							Time of the day with high mental performance						
	Morning		Evening			Feeling best in the morning		Morning		Evening			Morning mental performance	
***CLOCK 3111T/C***	**n**	**%**	**n**	**%**	***P*** ** value**	**OR (95% CI)**	***P*** ** value**	**n**	**%**	**n**	**%**	***P*** ** value**	**OR (95% CI)**	***P*** ** value**
*CC+TC*	225	43	109	53	**0.007**	0.684(0.491–0.953)	**0.024**	276	44	58	54	**0.028**	0.664(0.440–1.00)	**0.049**
*TT*	301	57	96	47				351	56	49	45			

ORs were calculated for combined groups of minor allele carriers (*CC+TC*) compared with *TT* subjects. Adjusted for sex, age and study site.

## Discussion

We initially detected a relationship between the *CLOCK 3111T/C* SNP and weight loss in a subset (n = 500) of the current population in which carriers of the minor allele (C) were less successful losing weight in response to a Mediterranean diet than their TT counterparts [8]. In the current study, we investigated factors underlying this association and demonstrated that these effects may be driven by behavioral factors and changes in ghrelin levels. Our results support the notion that the *CLOCK* locus influences behaviors related to weight loss, such as sleep reduction and evening preference, as well as appetite regulators such as plasma ghrelin levels.

Successful treatment of obesity represents a major health care challenge. Behavioral therapy has been proposed as part of weight loss programs. Its purpose is to facilitate the identification of triggers of inappropriate behavior conducive to weight gain, including excessive caloric intake and reduced physical activity [26]. Behavioral therapy is also used to develop appropriate responses based on the individual triggers [27]. Identification of loci involved in behavior may help to achieve more effective and individually tailored therapeutic strategies [28]–[30]. Our previous and current results show that the *CLOCK 3111T/C* SNP may contribute to this goal. Specifically, carriers of the minor allele *C* had more difficulty losing weight in the long term than *TT* carriers (3 kg difference).

It has been previously reported the *CLOCK 3111T/C* SNP may be functional through modification of mRNA stability and half life, thus influencing *CLOCK* translation and protein levels and affecting circadian rhythms and sleep duration [31]. Genotype-based differences in activity and sleep patterns have been shown in the context of bipolar disorders. Affected subjects and carriers of the minor *C* allele demonstrate a reduction in sleep duration which may affect the psychological and behavioral aspects of this mental illness [32], [33]. Likewise in our obese population, *CLOCK 3111C* carriers reported shorter sleep duration than *TT* carriers, and reduced sleep has reported to be obesogenic [34].

A number of mechanisms have been proposed to explain the association between reduced sleep and obesity [35]. One of them involves increased appetite resulting from alterations in leptin and/or ghrelin levels [36]. Alternatively short sleep duration could lead to weight gain by increasing the time available to eat [35]. Finally, it has been hypothesized that weight gain may be related to decreased energy expenditure resulting from increased fatigue and altered thermoregulation [35], [37].

With respect to the *CLOCK 3111T/C* variant, *C* carriers displayed significantly higher ghrelin plasma values than non-carriers, and this situation was particularly evident in the older group (≥38 ys). Moreover among *C* carriers, ghrelin was significantly and inversely correlated with weight loss. Epidemiological studies have shown a strong association between short sleep duration and higher ghrelin values. This mechanism activates neuropeptide Y (NPY)/agouti-related protein (AgRP) and inhibits proopiomelanocortin (POMC)/cocaine- and amphetamine-regulated transcript (CART) neurons, resulting in a net positive feeding signal [38]. Further support for the regulation of ghrelin by circadian clocks derived from mice models showed that in the absence of circadian clocks, ghrelin is no longer rhythmically expressed [39]. It remains to be determined how gastric clock genes regulate ghrelin synthesis and secretion; moreover, ghrelin plays a role in the anticipation of eating, including timing of meals, further affecting long-term regulation of body weight [40].

We did not find any relationship between leptin plasma values and the *CLOCK* SNP suggesting that this specific variant did not exert a significant effect on leptin regulation or, alternatively, that the specific characteristics of this population, all subjects in the overweight/obese range, prevented us from detecting associations that might be present in the general population.

Dietary habits could also be an underlying factor in the particular resistance of minor C allele carriers to losing weight. Evidence suggests that the Mediterranean Diet contributes to maintaining a healthy body weight [41]–[43]. In our study, *C* carriers of the *CLOCK3111 T/C* SNP showed a lower adherence to the Mediterranean Diet than *TT* subjects, and favored the consumption of protein, from animal sources and processed food, as suggested by the higher intake of trans fatty acids. This dietary pattern may be driving the less successful outcome of the weight loss program observed among the *C* allele carriers. In fact, olive oil consumed in the context of an energy balanced diet may facilitate weight control. Our group has shown that consumption of oleic acid is associated with lower adipocyte numbers in adipose tissue, suggesting that oleic acid intake may limit hyperplasia in obese subjects [44].

The other side of the energy balance is expenditure [35]. It has been reported that chronic sleep deprivation leads to morning tiredness and daylong feelings of fatigue that leads to reduced physical activity [45]. In the present study, the presence of the minor *C* allele was associated with reduced sleep and, consequently, with increased reporting of morning fatigue, lower mental performance in the morning and a preference for the evening hours. Moreover, among *C* allele carriers the evening preference was inversely correlated to physical activity in the current study, which was consistent with a previous report using objective measures of activity levels based on actigraphic devices [33], reinforcing our findings for the role of the *CLOCK* locus in sleep and behavioral preferences.

In conclusion, sleep reduction with changes in ghrelin values, alterations of eating behaviors and evening preference could be affecting weight and weight loss among *CLOCK 3111C* carriers. Our results support the hypothesis that the influence of the *CLOCK* gene may extend to a broad range of variables linked with human behaviors. Identifying *CLOCK* genotypes in patients may assist therapists in identifying the roots of the weight problem at the individual level and contribute to a more personalized and successful treatment.
